# Advances in GABA-Enriched Yogurt and Frozen Yogurt: Microbial Biosynthesis, Functional Properties, and Health Perspectives—A Comprehensive Review

**DOI:** 10.3390/foods14244254

**Published:** 2025-12-10

**Authors:** Muhammad Ameer Ushidee-Radzi, Chong Shin Yee, Raja Balqis Raja-Razali, Nur Asyiqin Zahia-Azizan, Tiziana Di Renzo, Anna Reale, Stefania Nazzaro, Pasquale Marena, Zul Ilham, Nur ‘Aliaa Abd Rahman, Wan Abd Al Qadr Imad Wan-Mohtar

**Affiliations:** 1 Functional Omics and Bioprocess Development Laboratory, Institute of Biological Sciences, Faculty of Science, University Malaya, Kuala Lumpur 50603, Malaysia; ameerushidee@um.edu.my (M.A.U.-R.); helenchong@um.edu.my (C.S.Y.); rajabalqislegit@gmail.com (R.B.R.-R.); asyiqinazizan@um.edu.my (N.A.Z.-A.); 2 Biomass Energy Laboratory, Faculty of Science, University Malaya, Kuala Lumpur 50603, Malaysia; 3 Institute of Food Sciences, National Research Council (CNR-ISA), Via Roma, 64, 83100 Avellino, Italy; anna.reale@isa.cnr.it (A.R.); stefania.nazzaro@isa.cnr.it (S.N.); pasquale.marena@isa.cnr.it (P.M.); 4 Environmental Science and Management Program, Institute of Biological Sciences, Faculty of Science, University Malaya, Kuala Lumpur 50603, Malaysia; ilham@um.edu.my; 5 Centre for Science and Environment Studies, Institute of Islamic Understanding Malaysia, 2 Langgak Tunku Off Jalan Tuanku Abdul Halim, Kuala Lumpur 50480, Malaysia; 6 Department of Process and Food Engineering, Faculty of Engineering, University Putra Malaysia, Serdang 43400, Malaysia; nuraliaa@upm.edu.my

**Keywords:** functional foods, fermented dairy products, probiotics, lactic acid bacteria, fermentation

## Abstract

Gamma-aminobutyric acid (GABA) is a bioactive, non-protein amino acid recognized for its role as an inhibitory neurotransmitter in the human central nervous system. Increasing interest in functional foods has increased attention on GABA due to its potential health benefits, including antihypertensive, anxiolytic, antidepressant, and neuroprotective effects. This review summarizes the natural dietary sources of GABA and explores advanced strategies for enriching dairy products, particularly yogurt and frozen yogurt (froyo), with GABA. Key microbial species capable of GABA biosynthesis via the glutamate decarboxylase (GAD) pathway are discussed, alongside enzymatic production techniques that support controlled GABA synthesis. A major focus of this review is the evaluation of various methods for incorporating GABA into dairy matrices, including direct GABA fortification and in situ fermentation using GABA-producing strains, with comparisons of yield, sensory attributes, and product stability. Physicochemical analyses and sensory evaluations are presented as essential tools for assessing product performance. Furthermore, the review outlines the therapeutic effects of GABA-fortified foods and their potential roles in managing hypertension, stress, and neurodegenerative disorders. Key challenges, including strain-dependent variability in GABA-production, storage stability, and regulatory compliance are addressed, along with market and legislative considerations for GABA-fortified foods. Future perspectives include the development of novel high GABA-producing strains, process optimization to improve product stability and sensory acceptance, and expanded applications within the functional food sector. Overall, this review provides an integrated, up-to-date overview of technological, functional and regulatory aspects, offering a clear scientific foundation for the development and commercialization of GABA-fortified dairy products.

## 1. Introduction

Gamma-aminobutyric acid (GABA) is a non-protein amino acid that functions predominantly as an inhibitory neurotransmitter in the mammalian central nervous system [1,2,3]. By binding primarily GABA-A and GABA-B receptors, it reduces neuronal excitability, maintaining a critical balance between excitation and inhibition [2,4]. More recently, GABA-C receptors, now often referred to as GABA-Aρ receptors, have been identified. These receptors possess ligand-gated ion channels similar to those of GABA-A receptors, although their precise physiological role in humans remains unclear [1,5]. The regulatory role of GABA is crucial in controlling processes such as anxiety, sleep regulation, and seizures modulation [5]. Endogenously, GABA is synthesized through the decarboxylation of glutamate by the glutamate decarboxylase enzyme (GAD), and its concentration is tightly regulated within neuronal tissues [1,2,4,5].

Beyond its neurological roles, GABA has gained attention for its potential health-promoting properties when consumed in the diet. Numerous studies have highlighted its antihypertensive, anxiolytic, antidepressant, antidiabetic, and immune-modulating effects [6,7,8,9,10,11]. The significance of GABA extends to the context of non-communicable diseases (NCDs) [12]. According to the World Health Organization, 3.7 million deaths were reported from NCDs in 2021, including cardiovascular diseases, diabetes, cancers, neurological disorders, chronic respiratory diseases, and digestive disorders [12]. Many of these diseases are increasingly prevalent across both developed and developing countries. For instance, hypertension and stress-related disorders are becoming increasingly prevalent in our modern society, and GABA-rich dietary interventions could offer a complementary approach to conventional treatments [13]. Additionally, GABA’s capacity to mitigate oxidative stress and neurodegeneration highlights its potential in managing age-related neurological conditions such as Alzheimer’s and Parkinson’s diseases [14,15,16]. The health benefits of consuming GABA-rich foods have been substantiated through numerous in vitro and in vivo studies. A study on GABA-enriched fermented milk demonstrated notable ACE inhibitory activity, contributing to its blood pressure-lowering effects [17]. Other studies have linked GABA intake to reduced stress and anxiety levels, improved sleep quality, and enhanced cognitive function [13,16,18].

**Figure 1:**
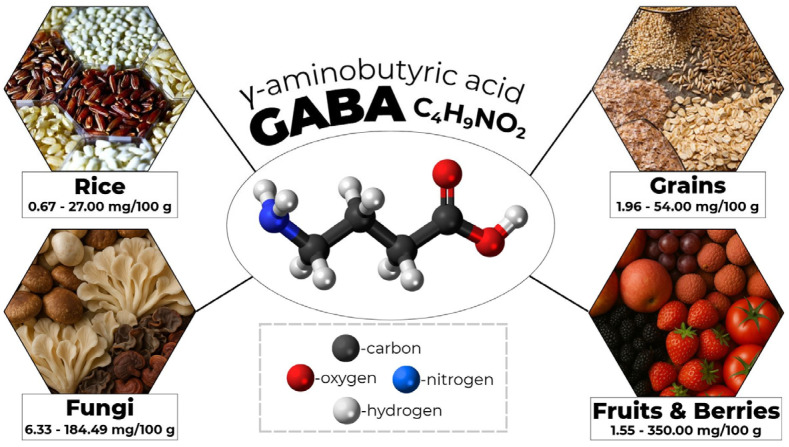
The most consumed plant-based foods containing GABA.

GABA is naturally present in a wide range of food sources [11] (Table 1). Numerous studies have shown that plant-based foods generally contain higher GABA levels than animal-derived foods, with concentrations reaching several mg g^−1^ depending on the plant species, developmental stage, and postharvest processing conditions (Table 1). Rice, grains, fungi, fruits and berries are among the most widely consumed GABA-rich foods globally (Figure 1).

As shown in Table 1, the GABA content of cereals and pseudocereals varies widely, from 0.67 mg/100 g in black rice to 54 mg/100 g in barley, depending on cultivar and processing conditions [11,19]. Among legumes, lupins are particularly noteworthy, reaching GABA concentrations of up to 46 mg/100 g. Fruits, berries, and vegetables also constitute important plant sources of GABA, with some varieties, such as lychees, containing up to 350 mg/100 g. Certain mushrooms, including the sun mushroom (*Agaricus brasiliensis*), have been reported to contain as much as 184.49 mg/100 g [11,57]. These variations reflect both intrinsic plant metabolic pathways and external factors such as cultivar selection, environmental conditions, and post-harvest handling. Although medicinal plants may also provide relevant GABA levels, their contribution to dietary intake is negligible due to the small quantities typically consumed. Other plant-derived products with exceptionally high GABA concentrations include pumpkin seeds (*Cucurbita pepo* and *Cucurbita maxima*), which may contain up to 1553 mg/100 g.

Animal-derived foods such as eggs and honey contain moderate amounts of GABA, whereas milk, regardless of the animal species, contains only a few micrograms per 100 mL. Moreover, GABA levels in these products are often substantially reduced during technological processing [31].

In contrast, both germination and fermentation significantly increase GABA content by activating glutamate decarboxylase (GAD), which enhances the conversion of glutamate into GABA [21]. Milk, in particular, represents an excellent substrate for producing a wide range of fermented dairy products. In recent years, substantial research has focused on microorganisms capable of synthesizing GABA, especially lactic acid bacteria (LAB) and yeasts, which provide safe and sustainable alternatives to chemical production methods. Among these microorganisms, lactobacilli are the predominant GABA producers in fermented foods with their efficiency largely determined by GAD activity and the availability of glutamic acid within the food matrix.

Because lactic acid fermentation can markedly elevate the natural GABA content, LAB strains with high GABA-producing capacity have been successfully employed to develop GABA-enriched fermented dairy products. Among these, yogurt and frozen yogurt stand out as ideal carriers due to their physicochemical stability, creamy texture, and sensory appeal, making them convenient vehicles for consistent intake of this health-promoting bioactive compound.

Based on these considerations, this comprehensive review provides an in-depth overview of the current literature on the production of yogurt and frozen yogurt enriched with bioactive GABA, as well as the key factors that influence GABA synthesis [66]. Relevant scientific articles were identified through systematic searches conducted in the ISI Web of Science, Scopus, and PubMed databases, covering publications from 2000 to 2025. Only articles published in English and focused on the nutritional, microbiological, and health-functional aspects of GABA-rich yogurt and frozen yogurt were included. After applying the inclusion criteria, the articles were screened by title and abstract, and duplicates were excluded. In total, 157 articles were deemed relevant to this review, highlighting the key challenges and opportunities related to the development and consumption of GABA-enriched yogurt and frozen yogurt, and contributing to a deeper understanding of their potential in promoting health-conscious and functional dietary patterns. This review also highlights methods used to characterize GABA-enriched yogurt and frozen yogurt, such as those reported by Li et al. [67], which include assessments of sensory attributes, functional properties, and therapeutic efficacy [67]. Moreover, the review aims to support the advancement of enhanced probiotic products—often described as “superfoods”—that deliver amplified health benefits through the synergistic action of probiotics and functional compounds like GABA. Emerging innovations, including the discovery of novel GABA-producing microbial strains and the use of artificial intelligence (AI) to optimize GABA biosynthesis, are discussed as promising future directions. A deeper understanding of GABA’s physiological roles, along with its broader integration into the food system, could play a significant role in advancing public health initiatives and improving dietary habits on a global scale.

## 2. Production Process for Yogurt and Frozen Yogurt

Yogurt (or Yoghurt) is a semi-solid dairy product produced through the fermentation of milk by thermophilic lactic acid bacteria (LAB) starter cultures. The primary bacteria used are *Streptococcus thermophilus* and *Lactobacillus delbrueckii* subsp. *bulgaricus*, though other species such as *Lactobacillus acidophilus*, *Bifidobacterium lactis*, *Lactococcus lactis*, and *Leuconostoc* spp. may also be involved [68,69,70]. *S. thermophilus* plays a crucial role as the initial producer of lactic acid, which lowers milk pH and creates a favorable environment for *L. delbrueckii* subsp. *bulgaricus* to grow [71]. Once active, *Lb. bulgaricus* breaks down milk casein into smaller peptides and amino acids, forming a gel-like network that traps water and gives yogurt its characteristic semi-solid texture. *S. thermophilus* and *Lb. delbrueckii* subsp. *bulgaricus* share a symbiotic relationship, in which each produces compounds that stimulate the growth and metabolic activity of the other. Together, they break down milk sugars and produce lactic acid creating a texture essential to yogurt [71]. Notably, this process reduces the lactose content, making yogurt more suitable for individuals with lactose intolerance who may otherwise struggle to consume dairy products in large amounts [70].

Aside from lactic acid, secondary metabolites are also produced within the process of fermentation of milk, which not only boost the nutritional profile of yogurt but also improve its digestibility. To mention a few, formates, acetaldehydes, or diacetyls that contribute to the fermented products’ aromatic, functional, nutritional, and textural qualities, as well as pyruvic acid, formic acid, acetone, and acetoin [70]. In addition to microbes, another important key in the production of yogurt is the milk itself. Yogurt is commonly made using cow’s milk; however, it may also be made using other types of milk such as goat’s milk, buffalo milk [72], and recently, with plant-based milk such as coconut milk [73], chickpea [74] as well as almond milk [75] and pistachio [76,77]. The trend towards creating plant-based yogurt is most likely to suit the dietary demands of people practicing a vegetarian and vegan diet as well as to enable those with intolerances such as lactose intolerance to enjoy the yogurt-like products [78].

Yogurts is a high-nutritious food, rich in essential vitamins and minerals such as vitamins B2 and B12 calcium, magnesium, potassium, and zinc, all of which are important for maintaining good health [68].

On the other hand, frozen yogurt, otherwise known as froyo or frogurt, is the frozen counterparts of yogurt, produced in a manner similar to ice cream [79,80]. It retains much of the original yogurt’s nutritional content and sensory attributes. A fermented frozen dairy product, frozen yogurt blends the nutritional value and flavor of fermented milk products with the texture of ice cream [79,81]. It’s a creative method to combine the benefits of ice cream with the therapeutic qualities of yogurt, making it a healthy substitute for ice cream for those with lactose intolerance, obesity and cardiovascular disorders, because it has less fat and lactose content than ice cream [82,83].

The primary difference lies in its texture and consistency. According to Hudson et. al. [79], frozen yogurt possesses a lighter, more aerated texture compared to traditional yogurt. In contrast to regular ice cream, frozen yogurt is typically slightly more acidic in flavor, reflecting its yogurt base [79]. Commercially, frozen yogurt is available in both soft-serve and hard-type forms [79]. Soft-serve frozen yogurt is usually freshly dispensed in stores for immediate consumption, whereas the hard-type version is prepackaged, offering longer shelf stability. They are commonly found in the freezer sections of grocery stores and other retail outlets.

**Figure 2:**
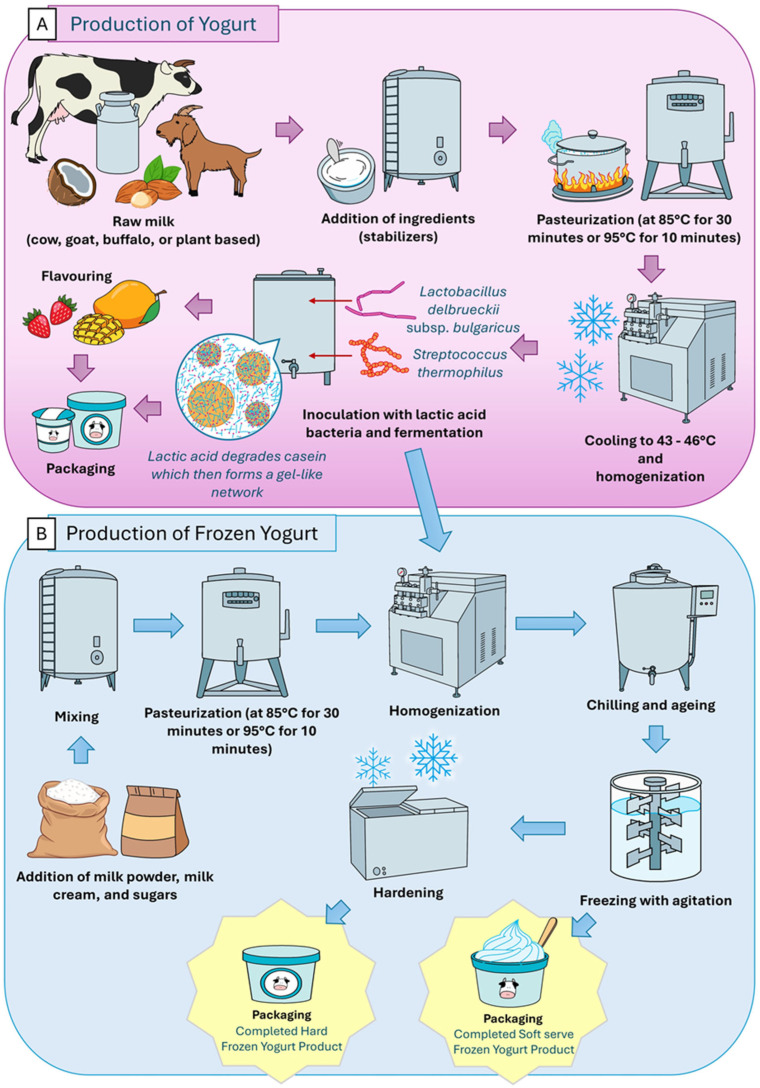
Production Process of Yogurt (**A**) and Frozen Yogurt (**B**).

The production processes of yogurt and frozen yogurt are closely interrelated, as shown in Figure 2. As previously stated, the process begins with the selection of milk. Different types of milk result in different types of yogurts, each with unique nutritional properties and health benefits.

One of the key characteristics that influence the final yogurt product is milk fat content. For instance, buffalo and goat milk contain significantly higher fat content (7.1% and 7.3%, respectively) compared to cow milk, which contains about 3.6% of fats [84]. Due to these variations, the type of milk used directly impacts the category of yogurt produced, typically classified as non-fat (NF), low-fat (LF) and high-fat (HF). These yogurt categories were defined by the United States Department of Agriculture (USDA) in 2001 [85]. According to these standards, HF must contain no less than 3.25% milk fat and at least 8.25% milk-solids-not-fat. Low-fat (LF) yogurt must contain between 0.5% and 2% milk fat, also with a minimum of 8.25% milk solids-not-fat. Finally, NF yogurt must contain no more than 0.5% milk fat while still maintaining at least 8.25% milk solids-not-fat [85]. Milk-solids-not-fat refer to all milk components excluding milk fat, like carbohydrates, starches, sugars, sweeteners, proteins, hydrocolloids, stabilizing agents and other dairy constituents [86,87]. The fat content within the yogurt is crucial in determining its quality and sensory characteristics. According to Erturk et al. [86], milk fat significantly contributes to flavor profile, mouthfeel, texture, whey syneresis (separation/expulsion of liquid), overall appearance, and physical properties of yogurt. Products with reduced fat content tend to perform poorly in many of these sensory attributes [86]. After milk selection, the next step in yogurt production is the addition of other ingredients. In most homemade or small-scale yogurt production, the process is straight-forward, typically involving milk and a yogurt starter culture or mother yogurt. However, in large-scale and commercial yogurt production, additional ingredients are often included to achieve specific product qualities and extend shelf life. These may include added fats, stabilizers [88] and occasionally, preservatives [89].

Proteins, too, are often added to yogurt to enhance its nutritional and textural qualities, with skimmed milk powder (SMP), as well as other whey and casein-based ingredients, being commonly used [88].

According to Arab et al. [88], increasing protein content not only improves the textural quality by enhancing gel elasticity but also helps reduce syneresis. However, each type of added protein comes with its own drawbacks. For instance, the use of SMP can result in a powdery mouthfeel, a strong fermented flavor, and a more intense acidic taste [88,90,91]. On the other hand, whey protein concentrates, isolates, hydrolysates, and other whey powders may lead to undesirable sensory attributes, such as a grainy texture and an unappealing whey flavor.

Adjustments of fat content in yogurt are typically performed by adding separated creams [91], such as cooking cream, heavy cream, double cream, and clotted cream. A combination of high fat content and proper homogenization results in the uniform distribution of small fat globules, which enhances yogurt water holding capacity. This, in turn, reduces syneresis and contributes to an improved texture and creamier mouthfeel.

Finally, the addition of stabilizers and preservatives is also somewhat common in commercial yogurt production. Stabilizers, particularly hydrocolloids such as carrageenans, carboxymethyl cellulose (CMC), sodium alginate and pectin, are added to achieve specific textural and physical properties in the final product. Each stabilizers serves a distinct purpose, for example, CMC is primarily used for its gel forming ability and its role in preventing syneresis. K-carrageenans also helps reduce syneresis, while sodium alginate functions mainly as thickening and emulsifying agent [87,92]. Due to the diverse functionalities of these compounds, the best practice to achieve a desired physical property of yogurt is via a mixture of different stabilizers with optimum proportions to each other. Preservatives such as potassium sorbate are added to extend shelf life [89]. This is particularly important in commercial production, allowing yogurt to remain stable during distribution and storage, both in retail environments and at home.

They primarily act as antimicrobial agents, inhibiting the growth of unwanted fungi, aerobic bacteria and yeasts [89]. However, because compounds such as potassium sorbate are usually synthesized through chemical processes, there has been growing interest in natural alternatives. Research has explored the use of plant-, algae-, and fungi-derived antioxidants as potential natural preservatives [89]. These are increasingly advocated as safer options, given the potential adverse health effects associated with synthetic preservatives [93].

Following the addition of ingredients, the yogurt premix undergoes pasteurization, a heat treatment process aimed to reduce microbial contamination. The specific temperature and duration depend on the method used. The most common approach is high-temperature pasteurization, where the premix is heated at 85 °C for 20–30 min or, alternatively, at 90–95 °C for 5 min for a shorter treatment. Other pasteurization techniques include low-temperature pasteurization carried out at 63–65 °C for 20 min, High temperature Short Time pasteurization at 72–75 °C for 15–20 s and Ultra High Temperature carried out at 145 °C for 1–2 s [71]. The process continues with the cooling and homogenization of yogurt mix. This is a relatively simple step, in which milk is cooled at 43–46 °C, for the next crucial stage: inoculation and fermentation. The inoculation process involves the addition of the main yogurt-making lactic acid bacteria *Lb. delbrueckii* subsp. *bulgaricus* (*Lb. bulgaricus*) and *S. thermophilus*, as well as any other desired functional or probiotic bacteria. This can be done by adding either a yogurt starter culture or “mother yogurt” from a previous batch [93]. Yogurt starter cultures are commonly available commercially in dried or powdered form, produced through the lyophilization (freeze-drying) of bacterial cultures grown in specific media. These commercial yogurt starters are generally food-grade, with some being made according to specific standards, including Kosher and Halal certifications [93]. As stated by Vedamuthu [93], several quality attributes are assessed in commercial yogurt starters before they reach the market. These include viable cell count, absence of contaminants and pathogens, functional activities (such as acid production), packaging integrity, accuracy of labeling, and compliance with declared shelf life. The use of commercial yogurt starters allows for greater precision and consistency, as they come with standardized data regarding bacterial composition and fermentation parameters, enabling producers to achieve specific yogurt characteristics within a controlled timeframe. On the other hand, mother yogurt is a more accessible option, as it refers to previously made yogurt that is used to inoculate a new batch with yogurt-making bacteria. In dairy plants, mother yogurt is often used to first inoculate an intermediate culture before being utilized to the final yogurt mixture. After inoculation, the yogurt mix undergoes fermentation, during which lactic acid bacteria (LAB) convert lactose into lactic acid—the key step in yogurt formation. Fermentation is typically carried out at 40–44 °C, which is the optimal growth temperature for *Lb. delbrueckii* subsp. *bulgaricus*. The duration of fermentation varies depending on the desired physical and sensory attributes, such as acidity, flavor, and texture. Once the desired level of fermentation is achieved, the process is halted by rapidly cooling the yogurt to approximately 4 °C. This drop in temperature significantly slows microbial activity without destroying the beneficial bacteria, thereby preserving yogurt flavor, texture, and, where applicable, probiotic content during storage, transport, and distribution [94].

The final step in yogurt production is packaging. The packaging material must be appropriate and chemically inert to prevent any reaction with yogurt, ensuring that product quality, safety, and shelf life are maintained. As brought forth by Saint-Eve et al. 2008 [94], concerns regarding yogurt packaging have been under investigation since 1992, beginning with research by Linssen et al. [95]. Their study revealed that aroma compounds in flavored drinking yogurts were being absorbed by polyethylene packaging, which compromised product sensory quality. Subsequent studies have since explored the broader effects of packaging materials on both the sensory and physicochemical properties of yogurt [94,95]. One significant finding relates to olfactory properties: among various packaging materials, glass exhibited the least aroma loss, followed by polystyrene and polypropylene [94]. Despite these insights, a need for more contemporary research focused on emerging packaging technologies remains, including active and environmentally friendly (green) packaging. These newer materials require further evaluation, particularly in the context of dairy products, which are typically stored for short durations and at low temperatures. Due to these specific conditions, the effects of packaging are often understudied or based on outdated model systems [96]. In addition to packaging, yogurt flavoring is a crucial step in the production process. Although flavoring may be introduced during the early stages of processing, it is generally preferred toward the end of fermentation. This timing helps ensure optimal development of the base yogurt, as early addition of flavoring agents or additives can interfere with microbial fermentation by altering parameters such as pH, which is critical for the activity of yogurt cultures [97]. Flavoring can involve the incorporation of whole fruits or the use of natural and artificial flavor compounds. Common fruits added to yogurt include strawberries, blueberries, raspberries, cane berries, peaches, cherries, and bananas. To enhance or replicate these fruity profiles, manufacturers may also use a variety of flavoring types—ranging from natural, to natural with other natural flavors, to natural and artificial, and fully artificial flavorings [98]. To improve visual appeal and suggest stronger flavor cues, colorants are frequently added to yogurt products. These may be incorporated directly or through fruit preparation steps. Any addition of color must be clearly declared on the product label with terms such as “Color added” or “Colored with _____,” specifying whether the colorant is natural or artificial.

In the case of frozen yogurt production, as shown in Figure 2, yogurt may immediately enter the frozen yogurt making process after the completion of the fermentation step, bypassing the need for packaging. If outsourcing is necessary, any externally produced yogurt may be utilized as a base for frozen yogurt production. The process typically begins with the incorporation of various additional ingredients such as milk (in pasteurized, raw, or powdered form), milk cream or creamer, sugars, stabilizers, emulsifiers, water, colorants, flavorings, and other optional ingredients such as toppings [79,83,99]. The specific formulation, including the ratio of ingredients, is determined by the manufacturer, based on the desired characteristics of the final product, such as texture, flavor, fat content, and the proportion of non-fat solids. All ingredients are commonly blended and homogenized together while being subjected to thermal treatment or pasteurization. This step is performed before adding the yogurt itself, in order to preserve the viability of the live bacterial cultures present in the yogurt. Once the mixture has sufficiently cooled, the yogurt is added and the mixture is homogenized again to ensure a smooth and uniform consistency.

Following the homogenization process, the mixture undergoes aging, a critical step in the production of both frozen yogurt and ice cream. As stated by Hudson et al. [79], aging is carried out at temperatures below 4 °C for at least 12 h. This step serves multiple purposes: it promotes fat crystallization, hydrates proteins and stabilizers (increasing viscosity of the mix), and facilitates the replacement of proteins on the membranes of fat globules with emulsifiers, an essential preparation for fat destabilization during freezing [79]. The final phase, prior to packaging, involves freezing and hardening, which is commonly carried out using a scraped surface heat exchanger or one of several types of freezers (continuous freezers, soft-serve freezers and batch freezers) operating at temperatures ranging from −2 °C to −6 °C [79,100]. The mixture must be agitated during freezing to introduce air, a process known as achieving overrun, which imparts the desired light and airy texture.

During this stage, the gel structure within the mixture is disrupted by agitation, thereafter partially reconstituting alongside the development of ice crystals during the hardening process, which imparts ice cream and frozen yogurt with a firm yet light and airy texture [79,100]. Once the frozen yogurt is churned, it is immediately discharged from the machine and collected either in its final packaging or in a temporary container. From there, it is swiftly transferred to cold storage to prevent melting and preserve quality. The finished product may be distributed and served as a soft-serve dessert, or stored at freezing temperatures in retail packaging as hard-frozen yogurt, similar to traditional ice cream. Yogurt and frozen yogurt (froyo) are widely consumed fermented dairy products recognized as excellent carriers for probiotics and bioactive compounds, including GABA. Their nutrient-rich composition, characterized by protein, fat, and lactose provides an ideal matrix for the survival and metabolic activity of beneficial probiotic microbes such as *Lactobacillus* and *Bifidobacterium* species [101,102,103]. The ability of these products to maintain a viable probiotic count during storage and gastrointestinal transit makes them suitable platforms for functional food development [104].

Probiotics play a dual role in yogurt and froyo production: they enhance gut health and act as natural biosynthesizers of bioactive compounds. Several strains, such as *Lactiplantibacillus plantarum*, *Levilactobacillus brevis*, and *Lactobacillus delbrueckii* ssp. *bulgaricus*, have demonstrated the ability to synthesize GABA during fermentation [105,106,107]. These microbes convert available glutamate into GABA via the glutamate decarboxylase (GAD) enzyme pathway, enabling in situ enrichment of the product.

Although froyo is traditionally consumed as a dessert, it has increasingly evolved into a functional food with potential health benefits. Unlike traditional yogurt, froyo is consumed in a frozen state, which poses unique challenges regarding probiotic viability and metabolic activity. However, studies have shown that, through optimized formulation, such as the use of cryoprotectants and cold-adapted strains, both probiotic survival and GABA retention can be preserved or even enhanced during frozen storage [106,108].

Yogurt and froyo, with their high sensory acceptability, convenience, and familiarity among consumers, represent ideal delivery systems for such bio-functional compounds. Moreover, the feasibility of GABA incorporation through either direct addition or microbial fermentation enhances the versatility and potential market of these dairy-based functional foods in addressing both nutritional and therapeutic goals [109].

## 3. GABA Production in Yogurt and Froyo

The increasing global interest in functional foods has brought gamma-aminobutyric acid (GABA) to the forefront of nutraceutical research. With its well-documented physiological benefits, including neuroprotective, antihypertensive, mood-regulating, and metabolic effects, GABA has become a promising candidate for incorporation into everyday consumables [11,109]. In response to this demand, different studies have explored the incorporation of GABA into widely consumed foods such as yogurt and frozen yogurt (froyo) with the aim to develop functional products that offer greater health benefits due to the synergistic effects of microorganisms, sometimes probiotics, and functional compounds such as GABA.

The microbial production of GABA as emerged as key biotechnological strategy for enhancing the functional properties of fermented foods, including yogurt and froyo. As highlighted by Di Renzo & Reale [110], advancements in microbial fermentation not only focus on better preserving foods but also on improving their sensory and nutritional qualities. Several microorganisms, particularly lactic acid bacteria (LAB) and certain fungi, possess the necessary enzymatic system to convert glutamate into GABA via the glutamate decarboxylase (GAD) pathway [106,111].

The primary biosynthetic route for GABA production, known as the GABA shunt pathway, takes place within the cytoplasm and cytosol of microbial cells. This pathway involves the decarboxylation of L-glutamate to GABA by the enzyme glutamate decarboxylase (GAD), which is typically expressed under acidic stress conditions [112,113].

In eukaryotic cells (e.g., fungi or yeast), GABA can be transported into mitochondria via GABA permease (GABP) for catabolism by the enzymes GABA transaminase (GABA-T) and succinic semialdehyde dehydrogenase (SSADH) to form succinate supplied to Krebs cycle or TCA cycle. Additionally, extracellular GABA may bind to GABA receptors under stress conditions [113], increasing intracellular Ca^2+^ levels, which in turn activate GAD and enhance cytosolic GABA production.

In bacterial strains, the GABA shunt operates entirely in the cytoplasm or associated with the cell membrane. Here, GABA contributes to cellular stress resistance and may be further catabolized within the cytoplasm without involvement of organelles [114].

A comprehensive overview of GABA-producing microorganisms is presented in Table 2, which highlights the variability in GABA yield among different microbial strains and fermentation substrates used.

*Lactobacillus* species are recognized as the most prominent microbial producers of GABA. *L. plantarum*, *L. brevis*, and *L. delbrueckii* ssp. *bulgaricus* have all shown to synthesize substantial quantities of GABA, especially under optimized fermentation conditions. For instance, *L. plantarum* L10–11, isolated from fermented fish (Plaa-som), was reported to produce a remarkable 15,740 mg/L of GABA in optimized media [115]. Similarly, *L. fermentum* strains from palm wine achieved GABA production levels up to 5340 mg/L [116], while *L. brevis* produces up to 150 mg/100 mL of GABA [107]. These findings highlight the potential of LAB strains as bio-fortifying agents in the development of GABA-enriched dairy matrices. In addition to bacterial sources, certain fungi strains, such as *Aspergillus oryzae* have also exhibited significant GABA biosynthetic capacity. Optimized *A. oryzae* strains, commonly used in soy sauce koji fermentation, have been show to produce over 3000 mg/L of GABA [117]. These fungi are particularly relevant in non-dairy fermentation systems and offer broader industrial potential due to their robust metabolic capabilities and adaptability to diverse substrates.

Key optimization strategies, including pH regulation, co-substrate supplementation, and targeted strain selection play critical roles in maximizing GABA production [10]. Importantly, the use of GRAS (Generally Recognized as Safe) organisms such as LAB ensures compliance with food safety regulations and enhances the commercial viability of GABA-enriched functional products. As the demand for natural health-enhancing foods grows, the identification and application of high-yield GABA-producing microorganisms will remain a cornerstone in the development of next-generation functional dairy products.

**Table 2:** GABA-producing microorganisms.

Microorganism	GABA Content	Source	References
*Aspergillus oryzae* NSK	451.70 mg/kg	Koji	[118]
*Aspergillus oryzae* NSK	194.00 mg/L	Soy sauce koji	[119]
*Aspergillus oryzae* NSK (production capacity optimisation)	236.74–354.08 mg/L	Soy sauce koji	[120]
*Aspergillus oryzae* NSK (production capacity optimisation)	3278.31 mg/L	Soy sauce koji	[117]
*Tetragenococcus halophilus* KBC	217.13–293.43 mg/L	Soy sauce *moromi*	[10]
*Tetragenococcus halophilus* KBC (production capacity optimisation)	653.1 mg/L	Soy sauce *moromi*	[10]
*Aspergillus oryzae* NSK and *Bacillus cereus* NSK (production capacity optimisation)	161.00 mg/L	Soy sauce koji and soy sauce *moromi*	[121]
*Aspergillus oryzae NSK* and *Tetragenococcus halophilus* KBC (production capacity optimisation)	159.00 mg/L	Soy sauce koji and soy sauce *moromi*	[121]
*Bacillus cereus* KBC	523.74 mg/L	Soy sauce *moromi*	[122]
*Bacillus cereus* KBC (production capacity optimisation)	3393.02 mg/L	Soy sauce *moromi*	[122]
*Lactobacillus delbrueckii* ssp. *bulgaricus* (production capacity optimisation) + other LAB species	36.1 mg/L	Yogurt	[10,105]
*Lactobacillus fermentum* (production capacity optimisation)	5340.0 mg/L	Palm wine	[116]
*Lactobacillus plantarum* L10-11 (production capacity optimisation)	15740.0 mg/L	Fermented fish (Plaa-som)	[10,115]
*Lactiplantibacillus plantarum* ssp. *plantarum* T-3	132.68 mg/L	Fermented cassava (*Growol*)	[123]
*Lactiplantibacillus plantarum* subsp. *plantarum* T-3 (production capacity optimisation)	164.95 mg/L	Fermented cassava (*Growol*)	[123]
*Lactobacillus plantarum* NDC75017 (production capacity optimisation)	314.56 mg/100 g	Traditional fermented chinese dairy products	[124]
*Lactobacillus plantarum* BC114	1450.0 mg/L	Chinese traditional paocai (*Sichuan paocai*)	[125]
*Saccharomyces cerevisiae* SC125	1030.0 mg/L	Chinese traditional paocai (*Sichuan paocai*)	[125]
*Saccharomyces cerevisiae* SC125 and *Lactobacillus plantarum* BC114	2420.0 mg/L	Chinese traditional paocai (*Sichuan paocai*)	[125]
*Lactiplantibacillus pentosus* SS6	55 mg/g	Fermented mulberry fruits	[126]
*Lactobacillus pentosus* 9D3	143.1 mg/L	Thai pickled weed	[127]
*Streptococcus salivarius* ssp. *thermophiles* fmb5	7300.0 mg/L	Direct Vat Set yogurt starter	[128]
*Streptococcus thermophilus* APC151	2000.0 mg/L	Digestive tract of fish (*L. mixtus*)	[129]
*Levilactobacillus brevis* F064A	3310.0 mg/L	Thai fermented sausage	[130]
*Levilactobacillus brevis* CGMCC 1.5954 (*L. brevis* 54)	1473.6 mg/L	Raw cow’s milk	[107]
*Lactobacillus brevis* Y8	61.3 mg/kg	Kimchi	[131]
*Pichia kudriavzevii* 1–21	614.0 mg/kg	Kazakh cheese	[132]
*Kluyveromyces marxianus* B13–5	956.0 mg/kg	Kazakh cheese	[132]
*Saccharomyces cerevisiae* DL6–20	450.0 mg/kg	Kazakh cheese	[132]
*Kluyveromyces lactis* DY1–10	793.0 mg/kg	Kazakh cheese	[132]
*Kluyveromyces marxianus* B13–5 and *Saccharomyces cerevisiae* DL6–20 (1:1)	189 mg/100 g	Kazakh cheese	[133]
*Pediococcus pentosaceus* HN8 and *Lactobacillus namurensis* NH2	4051 mg/kg	Fermented meats	[134]
*Lactococcus lactis* L-571	86.0 mg/L	Artisanal Mexican cheeses	[135]
*Lactococcus lactis* L-572	86.2 mg/L	Artisanal Mexican cheeses	[135]
*Lactococcus lactis* L-571 (production capacity optimisation)	1153.0 mg/L	Artisanal Mexican cheeses	[135]
*Lactococcus lactis* L-572 (production capacity optimisation)	140–200 mg/L	Artisanal Mexican cheeses	[135]
*Lactococcus lactis* L-571 and *Lactococcus lactis* L-572 (production capacity optimisation)	1147.0 mg/L	Artisanal Mexican cheeses	[135]

In the context of fermented dairy systems, such as yogurt and frozen yogurt (froyo), careful optimization of these parameters is essential for maximizing GABA yield. By fine-tuning fermentation conditions to align with the metabolic needs of selected microbial strains, producers can significantly enhance the functional properties of the final product. Glutamate, the precursor for GABA biosynthesis, is the principal substrate influencing GABA production. Microbial synthesis of GABA primarily depends on the activity of glutamate decarboxylase (GAD), which catalyzes the decarboxylation of glutamate into GABA [1,2,4,5].

GAD enzymes are predominantly derived from microorganisms, particularly lactic acid bacteria (LAB), fungi, and, to a lesser extent, plant systems. Microbial GADs, especially from *L. brevis*, *L. plantarum*, and *S. thermophilus*, have demonstrated high activity under food-compatible conditions [107,129,136,137]. Fungal species, including *Monascus* spp. and *Rhizopus* spp. have also been explored for their robust GAD activity in acidic environments, making them particularly suitable for GABA production in dairy matrices like yogurt and froyo [138,139].

Moreover, advancements in recombinant DNA technology have enabled the heterologous expression of GAD enzymes in hosts such as *Escherichia coli*. This allows for enzyme purification and industrial-scale production with enhanced stability and catalytic efficiency [140]. The availability of purified enzymes facilitates precise control over reaction conditions and minimizes the risk of microbial contamination, making it highly advantageous in food systems [140].

Both the presence and concentration of glutamate in the fermentation medium play a critical role in determining GABA yields. Several studies have shown that supplementation with monosodium glutamate (MSG) or glutamic acid can significantly enhance GABA production [141,142,143]. For instance, *L. brevis* demonstrated a marked increase in GABA yield upon MSG supplementation, often reaching levels over 1000 mg/L [144].

In natural dairy matrices, however, glutamate availability may be limited. Cow milk proteins, particularly β-casein, possess a high concentration of several metabolites such as valine, lysine, phenylalanine, lactate, 3-hydroxybutyrate and most importantly, glutamate [145]. Yet, the efficiency of GABA synthesis depends on the form of glutamate, whether it is present as a free amino acid or bound within peptide. Peptide-bound glutamate is less readily utilized by microbes for GABA production. To overcome this limitation, supplementation or pre-treatment methods (e.g., proteolysis) are often employed to release glutamate from milk proteins and enhance its bioavailability for microbial conversion into GABA [146].

Environmental conditions such as pH, temperature, oxygen levels, and incubation time substantially affect GABA biosynthesis. The optimal pH for GAD activity typically lies in the acidic range (around pH 4.0–5.5), aligning well with yogurt fermentation conditions [147]. Temperature is another key factor, with most LAB showing maximum GABA production between 30–37 °C [114,148]. Under suboptimal pH or temperature conditions, GAD activity is compromised, leading to reduced GABA accumulation.

Furthermore, anaerobic or microaerophilic environments generally favor both LAB growth and GABA synthesis. Studies on *L. delbrueckii* ssp. *bulgaricus* and *L. plantarum* have demonstrated that maintaining appropriate oxygen tension and incubation duration (24–48 h) is critical for maximizing GABA yields during dairy fermentations [105,109].

GABA production is closely linked to the microbial growth cycle, typically peaking during the late exponential to stationary phase. During this period, cells begin to accumulate stress-response metabolites, most likely due to the role of the GAD system in maintaining intracellular pH homeostasis and cellular integrity under acidic condition [149]. By monitoring and targeting this specific growth phase during fermentation, it is possible to enhance in situ GABA biosynthesis in products such as yogurt and froyo.

Maximizing GABA production in microbial systems requires a holistic understanding of microbial physiology and fermentation dynamics. A strategic optimization of all factors mentioned above is essential for developing efficient fermentation systems capable of producing GABA-enriched functional dairy products.

Enzymatic production of GABA provides an alternative to microbial fermentation and offers greater control over yield, purity, and reaction conditions [150]. This method involves the direct conversion of glutamate into γ-aminobutyric acid via the enzyme glutamate decarboxylase (GAD). GAD, a pyridoxal 5′-phosphate (PLP)-dependent enzyme, is responsible for catalyzing the decarboxylation of L-glutamic acid to GABA in a highly specific and efficient manner [111,149]. This was proven by Yogeswara et al. [150], who successfully synthesized GABA enzymatically from monosodium glutamate (MSG) using purified GAD, which was produced via recombinant DNA technology from *L. plantarum* FNCC 260, alongside PLP as cofactor [150].

PLP is essential as a coenzyme for the decarboxylation reaction, and its supplementation is often necessary for maximizing enzyme activity [114,147,148].

The substrate concentration also plays a pivotal role in determining GABA yield. Excess glutamate can increase GABA production [144], although substrate inhibition may occur at very high concentrations [122]. Therefore, a balance must be maintained to achieve maximum conversion efficiency. To improve process efficiency, enzyme immobilization techniques have been developed enhancing enzyme stability and reusability, allowing for continuous or repeated-batch production systems [151].

Compared to microbial fermentation, enzymatic production offers a faster and more predictable method for GABA synthesis, particularly in formulations requiring precise GABA enrichment. It is especially advantageous in contexts where microbial growth is limited by environmental conditions, such as in frozen yogurt or heat-processed dairy products. Nonetheless, the enzymatic synthesis of GABA may incur higher costs due to the need for additional processing steps, including enzyme production, isolation, and purification. Furthermore, despite improvements in enzyme immobilization, there remain challenges concerning enzyme stability and reusability, which can limit the overall economic feasibility when compared to microbial fermentation [13].

The incorporation of GABA into yogurt and froyo represents a promising strategy for developing functional dairy products aimed to support mental well-being and cardiovascular health [152]. GABA enrichment can be achieved through either direct addition of compound or through in-situ production during microbial fermentation. Each method offers distinct advantages and limitations, and the choice depends on the desired product characteristics, regulatory constraints, and consumer preferences.

Direct addition involves fortifying dairy products with purified GABA either during or after processing. A common approach is the supplementation with powdered GABA, typically produced by spray drying the fermentation broth of GABA producing microbes [13]. This approach offers precise control over the final GABA concentration and avoids variability associated with microbial fermentation. It also simplifies quality assurance and regulatory compliance, as the GABA content can be standardized and consistently monitored [109]. However, the stability of GABA under different processing and storage conditions is critical, especially in froyo where freezing may affect its bioavailability. In addition, flavor compatibility is essential, as excessive GABA addition might introduce off-tastes or negatively affect the sensory profile of the final product. Despite these limitations, direct addition remains a practical and scalable strategy, especially for commercial formulations targeting specific GABA dosages or application in non-fermented or heat-treated dairy matrices, where microbial viability and fermentation-based production may be limited.

In-situ production, instead, involves microorganisms synthesizing GABA during fermentation, aligning with clean-label trends and consumer demand for minimally processed, naturally functional foods. This method leverages GABA-producing bacteria such as *L. brevis*, *L. plantarum*, and *S. thermophilus*, which convert available glutamate, either naturally present or supplemented in milk, into GABA during yogurt or froyo fermentation [105,107,111,129].

Using starter cultures that naturally produce GABA allows for perfect integration of the compound during dairy production. For instance, *L. delbrueckii* ssp. *bulgaricus* and *S. thermophilus*, common yogurt starters, but also *L. plantarum* [124] can be selected or engineered to enhance GABA synthesis without compromising product quality and fermentation performances [129,153]. This method is really promising when probiotic microorganisms are incorporated simultaneously, providing a dual benefit: the generation of bioactive compounds like GABA, and the promotion of probiotic viability, both of which enhance the functional and commercial value of the final product [106,149].

Alternatively, hybrid approaches that combine both direct fortification and in-situ biosynthesis have recently gained attention. In these systems, yogurt or frozen yogurt is first fermented with GABA-producing starter cultures, followed by supplementation with additional purified GABA to achieve target concentrations [115,116]. Co-cultivation strategies allow the combination of desirable sensory traits from traditional starters with the functional GABA-enrichment capabilities of specialized strains. For instance, co-fermentation of species such as *Lactobacillus fermentum* SMN10-3(A) and *Lactococcus lactis* SMN15-6(B) has been shown to significantly increase GABA content in dairy matrices [154].

In a study conducted by Ramos et al. [155], 38 indigenous *Lactobacillus* strains, isolated from different food ecosystems, were assessed for GABA production capacity in sheep milk. They belonged to the species *Lactobacillus acidophilus*, *Levilactobacillus brevis*, *Lactobacillus delbrueckii*, *Lacticaseibacillus paracasei* and *Lactiplantibacillus plantarum*. Only two strains showed the highest GABA concentrations, approximately 200 mg/L, and milk derived from them showed higher viscosity and taste rating than the control [155].

This approach offers formulation flexibility and can be tailored to meet specific demographics target or health claims.

Incorporating GABA into yogurt and froyo, whether through direct fortification or fermentation-driven biosynthesis, expands the functional food landscape. These methods provide food technologists and manufacturers with versatile tools to develop innovative, health-oriented products that align with modern nutritional goals.

## 4. Qualitative Properties of GABA-Rich Yogurt and Frozen Yogurt

To ensure product quality, consumer acceptability, and therapeutic efficacy, GABA-rich yogurt and froyo must be thoroughly characterized in terms of their physicochemical, sensory, and stability attributes [141,153,156]. The incorporation of GABA, whether through fermentation or direct fortification, can influence parameters such as pH, viscosity, texture, flavor, and shelf life. Detailed characterization is essential for formulation optimization and for ensuring that functional claims meet both regulatory standards and consumer expectations.

**Figure 3:**
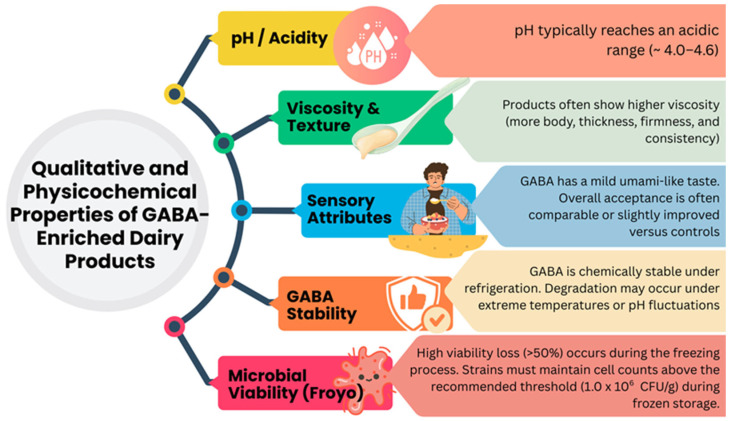
Qualitative aspects of GABA-enriched yogurt and frozen yogurt.

GABA production through microbial fermentation is often associated with acidification due to lactic acid accumulation, with a pH of yogurt and froyo to around 4.0–4.6 [66,107,136,148,149,157]. Interestingly, the GAD pathway may help microbes buffer intracellular pH under acidic conditions by consuming protons during glutamate decarboxylation [148,149]. Studies have shown that in yogurts fermented with *L. brevis* or *L. plantarum*, when the fermentation time increased, the pH gradually decreased and the amount of viable LAB increased, but GABA reached a maximum value [107,114,124,150]. In addition to pH, viscosity can be influenced by GABA-producing strains due to variations in exopolysaccharide (EPS) production and proteolytic activity (Figure 3).

Yogurt made with GABA-producing strains presented a higher viscosity and, as a result, more body, thickness, firmness, and consistency [66,129].

While some LAB strains enhance texture and mouthfeel through EPS synthesis, excessive proteolysis can weaken the protein matrix and reduce viscosity, underscoring the importance of optimized fermentation conditions [141,158,159]. Similarly, texture attributes, such as firmness, cohesiveness, and creaminess, are critical for consumer perception and must be preserved in GABA-enriched products [71,141]. This is particularly important for frozen yogurt, where freezing impacts ice crystal formation and protein network structure.

It has been claimed that more than 50% of viable bacterial counts are lost during the freezing methods used to make frozen sweets. Therefore, in order to ensure bacteria viability throughout freezing procedures of frozen dessert manufacture and storage, bacteria cell protection strategies such as immobilization, microencapsulation or prebiotics addition like inulin, or stabilizers like κ-carrageenan, iso-malt and corn starch could be required [81,87,92,97,106,108,151,160,161,162,163]. Studies have shown that co-fermentation with *L. plantarum* or supplementation with GABA does not negatively impact textural properties when properly formulated [141,164,165].

Sensory acceptance is key to the market success of GABA-rich dairy products. GABA itself has a mild umami-like taste and generally does not impart off-flavors when used at moderate concentrations [114,123].

However, fermentation byproducts, acidity, and texture changes can affect flavor and mouthfeel [124]. Sensory panel evaluations have reported comparable or slightly improved ratings in flavor balance, creaminess, and overall acceptability for GABA-enriched yogurts versus controls, particularly when optimized GABA-producing strains are used [105,111]. In general, look, odor, thickness, acidity, and fluidity of the mixed-starter culture GABA-rich dairy products and the control did not differ significantly. Therefore, the addition of GABA-producing strains for milk fermentation did not significantly affect sensory evaluation overall [124].

GABA does not degrade during fermentation, making it ideal for fortifying yoghurt. At moderate concentrations, it improves texture, rheology and sensory properties without negatively affecting overall perception. Higher concentrations worsen texture due to gel instability and high pH [166].

Regarding stability and shelf-life, GABA is chemically stable under refrigeration, although extreme temperatures or pH fluctuations can lead to degradation [141]. In frozen products, GABA levels have been shown to remain stable over several weeks if bacterial activity is sufficiently halted during storage. Furthermore, the viability of starter cultures is a concern in frozen system, but the use of cryoprotectants such as skim milk powder, inulin, or glycerol can help maintain microbial viability above the recommended threshold (10^6^ CFU/g) during frozen storage [78,80,167].

Specifically, during storage, the count remained below the initial value, indicating limited growth compared to inoculation, remaining within the recommended range of 10^6^–10^7^ CFU/mL, confirming that yogurt meets the requirements to be considered a probiotic food with potential health benefits [168]. Furthermore, no coliforms, molds, or yeasts were detected. Since the absence of these harmful microorganisms is essential to guarantee the product safety and general quality, this absence shows that the yogurt-frozen yogurt samples had excellent microbiological quality [51,168].

Overall, with appropriate strains selection, fermentation control, and ingredient synergy, GABA-enriched dairy products can achieve favorable physicochemical and sensory profiles while delivering scientifically supported health benefits.

## 5. Health Benefits and Applications

GABA-enriched functional foods have garnered heightened interest owing to their wide-ranging health benefits. Dairy-based products, such as yogurt and froyo, serve as effective delivery vehicles, providing a palatable and bioavailable means to consume GABA in amounts that exert physiological effects. As summarized in Table 3, various studies using in vitro assays, animal models, and clinical investigations have demonstrated the therapeutic potential of GABA in modulating blood pressure, alleviating anxiety and depression, and providing neuroprotective effects.

Based on Table 3, one of the most studied benefits of GABA is its antihypertensive effect. GABA lower blood pressure primarily by inhibiting angiotensin-converting enzyme (ACE), reducing vasoconstriction and promoting improved blood flow [170,171]. Several studies have validated this mechanism in functional food matrices. For instance, a GABA-enriched fermented milk exhibited significant ACE inhibitory activity (93 mU/mL) and effectively reduced blood pressure in animal models [17]. Further support comes from clinical trials, which confirm the blood pressure-lowering potential of GABA-fortified foods, specifically attributed to GABA (not to other components of milk such as calcium, magnesium or peptides), which acts peripherally by inhibiting the release of noradrenaline [176].

In a study of Nishimura et al. [25], the consumption of GABA-enriched white rice leds to significant reductions in both systolic and diastolic blood pressure in hypertensive adults. GABA plays a central role also in lowering hyperglycaemia, in improving gut microbiota in GABA soy fermented milk [177] and neuroinhibition, contributing to the regulation of stress and mood. This suggests synergistic effects between raw materials, GABA production, and bacteria. Its supplementation has been associated with reduced anxiety and alleviation of depressive symptoms, primarily by enhancing inhibitory signaling within the central nervous system [18,178]. Fermentation with *Lactobacillus* strains transforms products, making them healthy when enriched with high concentrations of GABA, which protects neuronal cells from toxins [175].

Several studies involving oral intake of GABA-enriched foods have reported beneficial outcomes, including reductions in cortisol levels, improvements in sleep quality, and increased alpha brainwave activity, which are indicators of relaxation [176,179]. GABA does not only act directly on the brain (as it may not easily cross the blood–brain barrier), but indirectly by regulating the gut microbiota, which influences the nervous system via the vagus nerve, neurotransmitters and short-chain fatty acids (SCFAs). This could open up avenues for natural treatments for insomnia, using probiotics and lactic ferments as a safe alternative to medication [173].

Actually, the incorporation of GABA in everyday foods like yogurt may serve as a dietary strategy to manage mild anxiety and stress-related conditions without pharmacological interventions. While the precise dosage-response relationship remains established in human populations, preclinical studies and pilot clinical trials on the anxiolytic and anti-depressant properties of GABA shows promising results [172].

Beyond its calming effects, GABA also exhibits neuroprotective properties. GABA-rich extracts have been shown to mitigate oxidative stress, reduce apoptosis, and enhance neuronal survival in cellular models exposed to toxic stimuli [180,181,182,183]. For instance, ethanolic extracts from germinated brown rice (GBR) containing GABA reduced mitochondrial membrane depolarization and phosphatidylserine translocation in SH-SY5Y cells, indicating protection against early apoptosis [180]. Similarly, a study using GABA-enriched chickpea milk demonstrated improved cell viability and reduced lactate dehydrogenase release in manganese-stressed PC12 cells [175]. These findings suggest that regular consumption of GABA-enriched foods could support brain health, particularly in aging populations or individuals at increased risk of neurodegenerative diseases.

Actually, the rising interest in natural nootropics (smart drugs), such as oxiracetam, and mood-supporting foods [184,185] has significantly expanded the market potential for GABA-enriched dairy products. Consumer surveys indicate an increasing demand for food products that support mental well-being and cardiovascular health [186].

In this evolving landscape, functional yogurts and frozen yogurts are well-positioned due to their familiarity, convenience, and broad demographic appeal. Analysis of market data reveals that the overall yogurt segment is expanding rapidly, providing a robust base for the integration of high-value nutraceuticals.

The yogurt category in particular is demonstrating vigorous expansion, with the market expected to increase from USD 2.9 billion in 2024 to USD 5.5 billion by 2034, registering a compound annual growth rate (CAGR) of 6.6% from 2025 to 2034 [187]. The frozen yoghurt market, on the other hand, although currently larger, is expanding at a slower pace, with growth from USD 3.6 billion in 2024 to USD 5.6 billion by 2034 and a projected CAGR of 4.6% [188]. Current consumption trends increasingly emphasize functionality, digestive wellness, and microbiome support. The yogurt category has evolved beyond simple probiotic claims to embrace prebiotics and postbiotics, creating a receptive environment for advanced functional ingredients. Within this context, GABA stands out as an ideal candidate. When incorporated through fermentation, GABA offers compounded benefits: viable lactic acid bacteria for gut health, additional physiological functions attributed to GABA, and the intrinsic nutrient density of dairy [141]. Integrating GABA into traditional yogurt aligns well with the category’s strong growth trajectory. With the GABA market projected to grow between 5.0% and 10.5% [189], adding it to a rapidly expanding yogurt base positions the product to shift from a simple daily staple to a targeted wellness solution.

Furthermore, as consumer preferences increasingly shift toward clean-label, minimally processed foods, the ability to generate GABA through natural fermentation confers an important competitive advantage. In-situ GABA biosynthesis allows manufacturers to meet both nutritional expectations and labeling preferences without relying on chemically synthesized additives, thereby reinforcing product transparency and consumer trust [190]. However, translating these opportunities into globally marketable products requires navigating complex and uneven regulatory landscapes. Regions differ widely in how they classify and authorize the use of novel or non-traditional functional ingredients. In the United States, GABA benefits from GRAS status, enabling its addition to a broad range of food products under established safety parameters. By contrast, the European Union adopts a more conservative stance, classifying GABA as a novel ingredient and imposing strict daily intake limits and stringent restrictions on health claims, which narrows the scope for marketing. Malaysia poses the most significant regulatory challenges. Here, GABA is not included within standard fortification schedules, and its use in food requires a formal petition process supported by extensive safety data, clear technological justification, and strict adherence to labeling rules, particularly those governing Quantitative Ingredient Declarations and additive classifications. Thus, in markets such as Malaysia, the major barrier to commercialization is not technical feasibility or consumer demand but regulatory approval procedures.

Taken together, while GABA-enriched yogurts and frozen yogurts hold strong commercial potential, supported by consumer trends, manufacturing feasibility, and growing international safety recognition, their long-term success will hinge on achieving an optimal balance between functional efficacy, sensory appeal, cost competitiveness, and regulatory compliance across diverse global markets.

## 6. Challenges and Future Perspectives

Despite the significant progress in developing GABA-enriched yogurt and froyo, several challenges remain in terms of optimizing production processes, ensuring consistency, and scaling for commercial success. Additionally, future research will need to address technological gaps, regulatory challenges, and evolving consumer expectations.

One of the primary challenges is ensuring consistent and high GABA yields during fermentation. Natural variability among microbial strains, fluctuations in raw material composition (e.g., milk or glutamate source), and environmental factors such as pH and temperature can affect GABA synthesis [106,111]. Furthermore, the selection of suitable starter cultures that simultaneously offer robust fermentation performance and high GABA productivity, without compromising sensory quality, remains a critical issue.

From a formulation standpoint, the stability of GABA during storage, particularly in frozen systems like froyo, must be controlled. Although GABA is chemically stable under cold conditions, microbial and enzymatic activity during storage could alter product composition. However, if the product is fortified and optimized, its antioxidant activity, sensory attributes, physicochemical characteristics, and microbiological stability of can be improved [191]. This modification may not influence the degradation of GABA; rather, it could potentially enhance GABA levels. Microbial activity during cold storage is generally reduced depending on the temperature, but residual activity may contribute to GABA production. Nonetheless, these ongoing processes raiser concerns regarding potential changes in product taste and texture. In fermented products, flavor, texture, and aroma are influenced by fermentation duration, potentially resulting in an unintended or excessively pronounced taste. Additionally, regulatory considerations for labeling and health claims differ between countries, affecting how GABA-enriched products can be marketed. For instance, while GABA is generally recognized as safe (GRAS) in many regions [192], claims regarding specific health benefits, such as blood pressure reduction or anxiety relief, typically require clinical substantiation.

The discovery and development of novel high-yield GABA-producing strains—including genetically enhanced or adaptively evolved variants—are keys to overcome yield bottlenecks. Advances in gene editing and strain engineering have enabled the identification of strains with significantly enhanced GABA-producing capabilities [193]. Genomic editing tools, notably the well-known CRISPR-Cas9, offer a powerful approach to genetically modify lactic acid bacteria including *Lactobacillus* spp., *Bifidobacterium* spp. and *S. thermophilus* [194,195,196,197]. Using CRISPR, even other bacterial species such as *E. coli* [198] can be engineered to exhibit enhanced GABA-producing. The potential applications are extensive: LAB strains with existing GABA-producing capacity can be optimized, and their functional genes can be transferred to other beneficial microorganisms via plasmids. This approach would enhance their trait and functionality, making them valuable in food industry. CRISPR can also improve fermentation properties of LABs, enabling precision engineering of metabolic pathways in generally recognized as safe (GRAS) microorganisms to efficiently utilize low-cost substrates for food production [199]. Beyond production efficiency, genome editing can enhance the sensory properties of fermented foods, including flavor, color, and texture [200,201,202]. While gene editing may be a futuristic solution for obtaining superior GABA-producing strains, exploring the biodiversity remains critical. Traditional fermented foods, unique microbiomes, and underutilized fermented products may harbor strains with exceptional GABA-producing potential [105,115]. The search for GABA-producing microorganisms dates back to the 1950s, when yeast extracts were first identified as GABA sources [106]. Since then, notable species, such as *L. fermentum* 4-17, isolated from kashkineh, an Iranian cereal based fermented food [168], have been identified, capable of producing up to 14.846 mg/100 mL of GABA [129].

Fermented foods may also harbor multiple GABA-producing bacterial species. For example, soy sauce has been used to isolate two distinct GABA-producing microbes: *Bacillus cereus* strain KBC, capable of producing 3393 mg/L of GABA, and *Tetragenococcus halophilus* strain KBC, which can produce 653.1 mg/L of GABA [10,121,122].

The isolation of GABA-producing microbes from food sources is particularly beneficial, as these microorganisms are already commonly consumed, which implies they are safe for human consumption. However, to establish their GRAS status, additional testing for pathogenicity and toxicity may be required to provide concrete evidence of their safety.

In addition to optimize microbial strain, enhancing fermentation techniques through bioprocess engineering, co-culturing methods, and precision fermentation can significantly boost GABA synthesis while maintaining desirable sensory and nutritional qualities [10]. One effective approach to optimize fermentation conditions is Response Surface Methodology (RSM), a statistical tool widely used to identify the ideal conditions, such as substrate volume, pH, temperature, inoculum percentage, aeration, and agitation speed, that maximize GABA yield [158]. Innovative techniques like two-stage fermentation (e.g., acidification followed by GABA conversion) or the use of immobilized cells can help decouple acid production from GABA biosynthesis, improving both yield and product quality [157]. Moreover, smart fermentation systems could also be researched and applied in order to maximize the production of GABA as well as other beneficial compounds in fermented foods. The integration of cutting-edge technologies such as biosensors, internet of things (IoT), artificial intelligence (AI) as well as machine learning may revolutionize the production of fermented foods, especially those rich in GABA. As highlighted by Yee et. al. [203], the incorporation of adaptive control systems into fermentative vessels can enable real-time monitoring of GABA production. These systems, using machine learning and AI, would adjust fermentation conditions dynamically based on the rate of GABA synthesis. For example, if its production begins to plateau due to substrate depletion, the system could automatically supply additional substrates to sustain optimal production levels. These smart fermentation systems could be applied in various feeding strategies, including continuous, batch, or fed-batch systems, enabling either batch-wise or continuous production of fermented foods.

The integration of these advanced technologies into fermentation processes could not only revolutionize the production of GABA-rich fermented foods but also transform the broader food industry. As a result, more nutraceuticals and superfoods—foods with health benefits beyond basic nutrition—could be developed, offering new opportunities for functional food production.

There is significant potential to expand GABA incorporation into a wider range of functional foods, extending beyond just yogurt and frozen yogurt (froyo). The development of GABA-enriched raw ingredients in food product formulations can help promote its diverse health benefits. Various raw food ingredients, such as rice, fruits, berries, and mushrooms, have been shown to naturally contain GABA [11], and these could be integrated into everyday foods. However, the use of natural ingredients may not be enough to create a truly GABA-rich product, as its content may diminish when mixed with other ingredients. To create a genuinely GABA-rich functional food, a product or dish could be developed using only GABA-rich raw materials. These could include smoothies, dairy-alternative beverages, and frozen desserts tailored to specific consumer groups, such as older adults or athletes who may benefit from increased GABA intake. For example, a smoothie traditionally made with ice cream could be reimagined with frozen yogurt as a GABA-rich alternative, incorporating GABA-rich fruits and berries like apples, mulberries, and grapes for natural flavoring. Recipes like these, using only GABA-enriched ingredients, would certainly meet the criteria of being classified as GABA-rich. However, if only foods do not appeal to general public, future formulations could combine its use with other bioactive compounds, such as prebiotics, polyphenols, or magnesium, to create synergistic health effects. These combinations could be supported by well-designed clinical trials to validate their benefits.

Furthermore, research into encapsulation technologies could enhance GABA stability and targeted delivery, improving its bioavailability and potentially masking any sensory drawbacks. For commercial success, interdisciplinary collaboration between food technologists, microbiologists, and marketing professionals will be crucial in moving GABA-based innovations from lab to market—turning concepts from the “bench to the shelf” or “bench to fork.” As mentioned above, the trend of creating plant-based yogurt is one of the emerging trends in food industry. As mentioned above, the trend towards creating plant-based yoghurts is one of the emerging trends in the food industry. This trend stems from the lifestyle choices of many people who practice vegetarianism and veganism. To meet the needs of these consumer groups, industries and researchers have turned to plant-based alternatives to dairy milk. These alternatives can be derived from a variety of plants, including coconut, soy, rice, oats, hemp, flax, peas and quinoa, as well as nuts such as almonds, pistachio and cashews [50,204].

The exploration of plant-based milks has paved the way for the development of plant-based yogurt alternatives, such as those made from soy milk [205], almond milk [75,206,207], quinoa [208], peanut milk [159], cashew milk and coconut cream or milk [73,207], pistachio [76,77,209,210], oat milk [211], chickpea [74], potato milk [174], and brown rice milk [127]. This research trend highlights the boom in phytoyogurt development, as industry continues to explore new plant-based yogurt varieties. These innovations pave the way for more inclusive options that cater to a wider range of dietary preferences, allowing individuals to enjoy the health benefits of probiotics and GABA, just like traditional dairy yogurt. As the market for plant-based foods continues to grow, these dairy-free alternatives could become a mainstream option for people seeking both nutritional benefits and inclusivity.

## 7. Conclusions

Incorporating GABA into yogurt and frozen yogurt represents a compelling intersection of food science and preventive health. Through microbial or enzymatic strategies, GABA can be efficiently produced and retained in dairy matrices, enabling the development of functional foods with tangible physiological benefits. The well-documented health advantages of GABA, including cardiovascular regulation, improved mental well-being, and neuroprotection, highlight its potential as a dietary intervention for modern lifestyle-associated disorders. Although challenges remain in optimizing microbial strains, maintaining sensory quality, and securing regulatory approval, advancements in fermentation technology and bioprocessing are offering promising solutions. Continued exploration of high-GABA-producing strains, smart fermentation systems, and synergistic bioactive formulations holds the key to future innovation in this field. Ultimately, GABA-enriched dairy products stand as practical and appealing vehicles for delivering scientifically supported health benefits to a broad consumer base. With ongoing research and innovation in phyto-yogurts enriched with GABA, these products have the potential to make a meaningful contribution to public health, addressing issues such as stress, cardiovascular risk, and neurodegeneration, while remaining both accessible and enjoyable for all.

## Figures and Tables

**Table 1:** GABA content in different raw materials.

Raw Materials	Varieties	Content		References
		Lowest	Highest	
Rice	Black rice (*Oryza sativa* L.)	0.67 mg/100 g	7.46 mg/100 g	[11,19,20]
	Brown rice (*Oryza sativa* L.)	5.28 mg/100 g	27.00 mg/100 g	[11,21,22]
	Red rice (*Oryza sativa* L.)	1.18 mg/100 g	2.91 mg/100 g	[11,23,24]
	White rice (*Oryza sativa* ‘Yumepirika’)	2.70 mg/100 g	11.20 mg/100 g	[25]
Grains	Barley (*Hordeum vulgare* L.)	1.96 mg/100 g	54.00 mg/100 g	[11,26]
	Corn (*Zea mays* L.)	-	15.27 mg/100 g	[11,27]
	Kodo millet (*Paspalum scrobiculatum*)	-	7.15 mg/100 g	[11,28]
	Wheat (*Triticum aestivum* L.)	4.55 mg/100 g	14.68 mg/100 g	[11,29]
	Quinoa (*Chenopodium Quinoa*)	-	10.45 mg/100 g DW	[30,31]
	Chia (*Salvia hispanica* L.)	-	9.51 mg/100 g	[31,32]
	Buckwheat (*Fagopyrum esculentum*)	-	12.4 mg/100 g DW	[31,33]
	Amaranth (*Amaranthus caudatus* L.)	-	2.6 mg/100 g DW	[27,31]
Legumes	Lentil (*Lens culinaris*)	-	10.37 mg/100 g DW	[30,31]
	Chickpea (*Cicer arietinum*)	-	8.7 mg/100 g DW	[30,31]
	Soybean (*Glycine max*)	-	25 mg/100 g	[31,34]
	Adzuki bean seeds (*Vigna radiata* L.)	-	21.31 mg/100 g	[31,35]
	Lupin *(Lupinus angustifolius* L. var. *zapaton*)	-	46 mg/100 g	[31,34]
	Peas (*Pisum sativum*)	-	1.5 mg/100 g	[31,36]
Fruits & Berries	Apple (*Malus pumila* Mill.)	-	9.71 mg/100 g	[11,37]
	Peach (*Prunus persica* L. Batsch)	-	0.008 mg/mL	[31,38]
	Grape (*Vitis vinifera* L.)	5.89 mg/100 g	10.98 mg/100 g	[11,39]
	Lychee (*Litchi chinensis* Sonn.)	170.00 mg/100 g	350.00 mg/100 g	[11,40]
	Mulberry fruit (*Morus alba* L.)	17.10 mg/100 g	33.60 mg/100 g	[11,41]
	Raspberry (*Rubus idaeus*)	-	10.1 mg/100 g FW	[31,42]
	Black Raspberry (*Rubus occidentalis*)	-	19.4 mg/100 g FW	[31,42]
	Strawberry (*Fragaria × ananassa* Duch.)	1.55 mg/100 g	3.61 mg/100 g	[11,43]
	Blueberry (*Vaccinium corymbosum*)	7.9 mg/100 g FW	8.9 mg/100 g FW	[31,42]
	Kiwi (*Actinidia deliciosa*)	8 mg/100 g	14 mg/100 g	[31,44]
	Jujube (*Zizyphusju-juba* Mill.)	-	140 mg/100 g DW	[31,45]
	Longan (*Dimocarpus longan* Lour.)	134 mg/100 g	144 mg/100 g	[31,46]
	Tomato (*Solanum lycopersicum* L.)	21.50 mg/100 g	189.7 mg/100 g	[47]
Vegetables	Carrot *Daucus carota* subsp. *sativus*)	0.014–0.3 mg/100 g DW	230–280 mg/100 g DW	[31,48,49,50]
	Bitter melon (*Momordica charantia* L.)	-	283.8 mg/100 g DW	[31,51]
	Broccoli *(Brassica oleracea* var. *italica*)	-	12.88 mg/100 g FW	[30,31]
	Potato (*Solanum tuberosum*)	-	44.86 mg/100 g FW	[30,31]
	Parsley (*Petroselinum crispum*)	-	28.18 mg/100 g FW	[30,31]
	Red beet (*Beta vulgaris*)	-	18,84 mg/100 g FW	[30,31]
	Asparagus (*Asparagus officinalis* L.)	-	16–111.6 mg/100 g DW	[31,52]
	Red mustard flower buds (*Brassica juncea* (L.) *Czern*)	-	179.8 mg/100 g FW	[31,53]
	Spinach (*Spinacia oleracea*)	-	4.3 mg/100 g	[31,49]
	Zucchini (*Cucurbita pepo* L)	1.5 mg/100 g	4 mg/100 g	[31,54]
	Brussel Sprouts seeds (*Brassica oleracea*)	62.75 mg/100 g	70.61 mg/100 g	[31,55]
Fungi	White mushroom (*Agaricus bisporus*)	18.00 mg/100 g	20.00 mg/100 g	[11,56]
	Sun mushroom (*Agaricus brasiliensis*)	-	184.49 mg/100 g	[57]
	Shiitake mushroom (*Lentinula edodes*)	17.00 mg/100 g	35.00 mg/100 g	[11,58]
	Oyster mushroom (*Pleurotus pulmonarius*)	32.15 mg/100 g	57.73 mg/100 g	[11,59]
	Wood ear mushroom (*Auricularia polytricha*)	-	28.16 mg/100 g	[57]
	Baby lingzhi (*Ganoderma lucidum*)	-	6.33 mg/100 g	[57]
Medical plants	Bistort root (*Bistorta officinalis*)	-	57.3 mg/100 g DW	[30,31]
	Chamomile flower (*Matricaria chamomilla*)	-	51.4 mg/100 g DW	[30,31]
	Lophanthus, aerial parts (*Lophanthus chinensis*)	-	49.3 mg/100 g DW	[30,31]
	Basil, leaf (*Ocimum basilicum*)	-	26.9 mg/100 g DW	[30,31]
	White oregano leaf (*Origanum heracleoticum*)	-	23.8 mg/100 g DW	[30,31]
	Lemon balm leaf (*Melissa officinalis*)	-	21.6 mg/100 g DW	[30,31]
	Mint leaf (*Mentha piperita*)	-	19.4 mg/100 g DW	[30,31]
	Salvia leaf (*Salvia officinalis*)	-	17.2 mg/100 g DW	[30,31]
	Thyme aerial parts (*Thymus vulgaris*)	-	15.9 mg/100 g DW	[30,31]
	Rosemary aerial parts (*Rosmarinus officinalis*)	-	13.7 mg/100 g DW	[30,31]
	Lavender flower (*Lavandula angustifolia*)	-	12.5 mg/100 g DW	[30,31]
Others	Cocoa beans (*Theobroma cacao* L)	31.7 mg/100 g	101.2 mg/100 g	[31,60]
	Pumpkin seeds (*Cucurbita pepo* and *Cucurbita maxima*)	371 mg/100 g	1553 mg/100 g	[31,61]
	Broccoli seeds (*Brassica oleracea* var. *italica*)	104.1 mg/100 g	108.9 mg/100 g	[31,55]
Animal products	Human milk	-	1 µg/100 mL	[31,62]
	Cow milk	-	1.4 µg/100 mL	[31,62]
	Goat milk	-	6.2 µg/100 mL	[31,62]
	Camel milk	-	~7 µg/100 mL	[31,62]
	Honey	0.6 mg/100 g	61.5 mg/100 g	[31,63,64]
	Egg yolk	5.77 mg/100 g	14.37 mg/100 g	[31,65]

**Table 3:** In vivo studies on health benefits of GABA-enriched foods.

Health Benefits	Ingredients	Model	Doses	GABA Content	Key Findings	References
Anti-hypertensive effect	GABA-enriched yogurt containing *L. plantarum* Taj-Apis362 + starter culture (*S. thermophilus* + *L. delbrueckii* ssp. *bulgaricus*)	Spontaneously hypertensive rats (SHR)	Three doses of yogurt (30, 150, 300 mg/kg)	59 mg/100 g	No significant difference between GABA doses (*p* > 0.05), indicating that 30 mg/kg yogurt (0.1 mg/kg GABA) is effective.	[169]
	GABA-enriched milk fermented with *Lacticaseibacillus casei* strain Shirota and *Lc. lactis* YIT 2027 (FMG)	39 mildly hypertensive patients aged 28–81 years	100 mL of FMG daily at breakfast for 12 weeks + 2 weeks of no intake	10–12 mg/100 mL	FMG reduces BP in patients with mild hypertension (grade 1–2 WHO/ISH) without altering heart rate or causing side effects, with a dose-dependent effect from GABA (10–12 mg/day). The effect is rapid (within 2–4 weeks), persistent during intake and partially reversible after discontinuation.	[170]
	GABA-enriched milk fermented with *Lb. casei* strain Shirota and *Lc. lactis* YIT 2027 (FMG)	SHR and normotensive Wistar–Kyoto (WKY/Izm) rats	Single oral dose of FMG (experiment 1) + chronic oral dose (3 weeks) (experiment 2)	0.5 mg/kg (experiment 1) 0.1 mg/kg (experiment 2)	Experiment 1 = In hypertensive rats (SHR), systolic blood pressure was significantly reduced after 4–8 h. The effect disappeared after 24 h. No change was observed in normotensive rats (WKY) → the effect is selective for those with hypertension. Experiment 2 = delayed increase in SBP in hypertensive rats.	[171]
	GABA-enriched fermented milk with *Lacticaseibacillus paracasei* spp. *paracasei* NTU 101 (101FM) and *Lactobacillus plantarum* NTU 102 (102FM), separately	SHR	Single oral dose of FMG + 101FM and FMG + 102FM (experiment 1) + chronic oral dose FMG + 101FM and FMG + 102FM (8 weeks) (experiment 2)	970 mg/L	101FM significantly reduced systolic (SBP) and diastolic (DBP) blood pressure as early as 4 h, maintaining the effect for up to 24 h. 102FM and GABA showed significant reductions, especially at 8 h (experiment 1) Both (101FM and 102FM) slowed the increase in blood pressure in SHR rats compared to the control group (experiment 2).	[17]
	GABA enriched-white rice	39 adults (aged 40–64 years) with mild hypertension	150 g rice for 8 weeks	11.2 mg GABA per 100 g of rice	Morning systolic blood pressure decreased significantly, starting in week 1 and markedly in weeks 6 and 8 (and also 1 week after the end of the study). Morning diastolic blood pressure improved in week 1 post-study.	[25]
Anxiolytic effect	GABA-enriched yogurt with *Levilactobacillus brevis* CGMCC1.5954 + starter culture (*S. thermophilus* ABT-T and *L. bulgaricus* BNCC 336436)	56 male mice with circadian rhythm disorders	0.4 mL of GABA aqueous solution per day (0.33 g/L (low-dose), 0.65 g/L (medium-dose), and 1.30 g/L (high-dose))	147.36 mg/100 mL	Intake of high-dose GABA-enriched yogurt group was effective in reducing levels of oxidative stress, 5-HT and Glu in mince brain tissue and increased serum GABA levels, resulting in the alleviation of symptoms related to anxiety and memory loss in mice.	[172]
Anti-insomnia effect	GABA-enriched fermented milk with *Levilactobacillus brevis* DL1-11	Sixty mice divided into groups: control, fermented milk without GABA, low/medium/high doses of milk with GABA, and a group with diazepam (an anti-anxiety drug as a comparison).	Oral daily dose (30 days)	LGFM group (low-dose GABA fermented milk): 8.83 mg/kg MGFM group (medium-dose GABA fermented milk): 16.67 mg/kg HGFM group (high-dose GABA fermented milk): 33.33 mg/kg	Milk with high doses of GABA reduces anxiety and improves sleep (longer sleep and reduced latency). No significant effect with low doses or without GABA.	[173]
Improvement of hyperglycaemia and balance of the gut microbiota	GABA-soy milk fermented with high GABA-producing *L. plantarum* GA30 (LPGA30) and low GABA-producing *L. plantarum* PV30 (LPPV30)	STZ-induced hyperglycaemic mice	Daily oral dose	30 mg/g	High GABA-producing *L. plantarum* GA30 yogurt improves glucose regulation, restores β-cell insulin production and rebalances gut microbiota with beneficial microbes.	[174]
Neuroprotective effect	GABA-enriched chickpea milk fermented with *L. plantarum* M-6	PC12 cells MnCl2-injured	500, 1000, 1500, 2000 μg/mL	17.78 mg/g	Improvement of cell viability, markedly attenuation of lactate dehydrogenase release and recovery of cell morphology	[175]

## Data Availability

No new data were created or analyzed in this study. Data sharing is not applicable to this article.

## Abbreviations

The following abbreviations are used in this manuscript:

ACE	Angiotensin-converting enzyme
AI	Artificial Intelligence
CFUs	Colony forming units
CMC	carboxymethyl cellulose
CRISPR	Clustered Regularly Interspaced Short Palindromic Repeats
DNA	Deoxyribonucleic acid
EFSA	European Food Safety Authority
GABA	Gamma-aminobutyric acid
GABA-T	GABA transaminase
GABP	GABA permease
GAD	Glutamate decarboxylase
GRAS	Generally recognized as safe
HF	High fat
IoT	Internet of Things
LAB	Lactic acid Bacteria
LF	Low fat
MSG	Monosodium glutamate
NCD	Non-communicable diseases
NF	Non-fat
PBMA	Plant-based milk Alternatives
PLP	Pyridoxal 5′-phosphate
RSM	Response Surface Methodology
SHR	Spontaneously hypertensive rats
SMP	Skimmed milk powder
SSADH	Succinic semialdehyde dehydrogenase
TCA	Tricarboxylic Acid
USDA	United States Department of Agriculture
